# 1,25-Dihydroxyvitamin D_3_ modulates calcium transport in goat mammary epithelial cells in a dose- and energy-dependent manner

**DOI:** 10.1186/s40104-016-0101-0

**Published:** 2016-07-28

**Authors:** Feifei Sun, Yangchun Cao, Chao Yu, Xiaoshi Wei, Junhu Yao

**Affiliations:** College of Animal Science and Technology, Northwest A&F University, Yangling, 712100 Shaanxi Peoples Republic of China

**Keywords:** Calcium, Dairy goat, Glucose, Transport, Vitamin D

## Abstract

**Background:**

Calcium is a vital mineral and an indispensable component of milk for ruminants. The regulation of transcellular calcium transport by 1,25-dihydroxyvitamin D_3_ (1,25-(OH)_2_D_3_, the active form of vitamin D) has been confirmed in humans and rodents, and regulators, including vitamin D receptor (VDR), calcium binding protein D_9k_ (calbindin-D_9k_), plasma membrane Ca^2+^-ATPase 1b (PMCA1b), PMAC2b and Orai1, are involved in this process. However, it is still unclear whether 1,25-(OH)_2_D_3_ could stimulate calcium transport in the ruminant mammary gland. The present trials were conducted to study the effect of 1,25-(OH)_2_D_3_ supplementation and energy availability on the expression of genes and proteins related to calcium secretion in goat mammary epithelial cells.

**Methods:**

An in vitro culture method for goat secreting mammary epithelial cells was successfully established. The cells were treated with different doses of 1,25-(OH)_2_D_3_ (0, 0.1, 1.0, 10.0 and 100.0 nmol/L) for calcium transport research, followed by a 3-bromopyruvate (3-BrPA, an inhibitor of glucose metabolism) treatment to determine its dependence on glucose availability. Cell proliferation ratios, glucose consumption and enzyme activities were measured with commercial kits, and real-time quantitative polymerase chain reaction (RT-qPCR), and western blots were used to determine the expression of genes and proteins associated with mammary calcium transport in dairy goats, respectively.

**Results:**

1,25-(OH)_2_D_3_ promoted cell proliferation and the expression of genes involved in calcium transport in a dose-dependent manner when the concentration did not exceed 10.0 nmol/L. In addition, 100.0 nmol/L 1,25-(OH)_2_D_3_ inhibited cell proliferation and the expression of associated genes compared with the 10.0 nmol/L treatment. The inhibition of hexokinase 2 (HK2), a rate-limiting enzyme in glucose metabolism, decreased the expression of PMCA1b and PMCA2b at the mRNA and protein levels as well as the transcription of Orai1, indicating that glucose availability was required for goat mammary calcium transport. The optimal concentration of 1,25-(OH)_2_D_3_ that facilitated calcium transport in this study was 10.0 nmol/L.

**Conclusions:**

Supplementation with 1,25-(OH)_2_D_3_ influenced cell proliferation and regulated the expression of calcium transport modulators in a dose- and energy-dependent manner, thereby highlighting the role of 1,25-(OH)_2_D_3_ as an efficacious regulatory agent that produces calcium-enriched milk in ruminants when a suitable energy status was guaranteed.

## Background

As a crucial macro-mineral for animals, calcium has functions in many physiological processes, including skeletal formation, nerve pulse transmission, muscle contraction, blood clotting, stimulus secretion coupling, and is an indispensable component of milk [1–3]. Milk is a naturally calcium-rich fluid produced by animals and humans. Actually, the total calcium concentration in ruminant milk is approximately 30 mmol/L [4]. It was reported that a substantial calcium flux was generated from blood to milk during the lactation period [5–8]. Accordingly, there must be a precise regulatory mechanism involved in the modulation of calcium transport in the mammary glands of dairy animals.

It is not entirely understood how mammary epithelial cells (MECs) extract large quantities of ionized calcium from plasma and produce a calcium-rich secretion, particularly for ruminants. The blood total calcium levels of dairy cows have a narrow range (approximately 2.0 to 2.5 mmol/L) [8]; thus, the process of calcium transport in the mammary gland occurs against a tremendous concentration gradient. Moreover, VanHouten and Wysolmerski [9] reported the existence of transcellular calcium transport and summarized this process in human MECs. Consequently, it can be extrapolated that the transcellular process is involved in calcium transport during milk secretion in ruminants.

Calcium-transport proteins, such as calcium binding protein-D_9k_ (calbindin-D_9k_), plasma membrane Ca^2+^-ATPase 1b (PMCA1b) and 2b (PMCA2b), have been confirmed as essential elements for transcellular calcium transport [5, 7, 10, 11]. According to recent research, Orai1, a pore subunit of the Ca^2+^ release-activated Ca^2+^ (CRAC) channels, is essential for calcium entry into cells and calcium homeostasis [12–14], but no trial has been conducted in mammary epithelial cells from dairy goats. Evidence circumstantiated that 1,25-dihydroxyvitamin D_3_ (1,25-(OH)_2_D_3_), the active form of vitamin D, was the most critical regulator of transcellular calcium transport and body calcium homeostasis [1, 15, 16]. 1,25-(OH)_2_D_3_ stimulated mammary calcium transport to elevate the milk calcium content by upregulating calbindin-D_9k_ and PMCA2b in lactating mice; knockout mice were used in this study [17]. Furthermore, 1,25-(OH)_2_D_3_ has been reported to facilitate the synthesis of epithelial calcium channels, increase the expression of plasma membrane calcium pumps, and induce the formation of calbindin in humans, rats and other species [18–20]. In addition, Kohler et al. [21] measured the blood concentrations of 1,25-(OH)_2_D_3_ in lactating goats at different altitudes, but the potential regulatory effects of 1,25-(OH)_2_D_3_ on mammary calcium transport and milk secretion, such as the expression of key regulators, were not studied. In summary, few research studies called attention to goat mammary calcium transport, and it has not been fully elucidated whether 1,25-(OH)_2_D_3_ regulates calcium transport in goat MECs.

Therefore, we hypothesized that 1,25-(OH)_2_D_3_ supplementation could modulate the expression of genes involved in calcium transport in goat MECs in a dose-dependent manner. Meanwhile, as an active transport process, calcium transport might be influenced by the cellular energy status.

## Methods

### Ethics statement

In the present research, all the procedures and operation were approved by the Animal Welfare Committee of Institute of Animal Nutrition and Feed Science, College of Animal Science and Technology, Northwest A&F University, Yangling, Shaanxi, P.R. China.

### In vitro culture of goat mammary epithelial cells

Dulbecco’s Modified Eagle Medium F12 (DMEM/F-12), fetal bovine serum (FBS), epidermal growth factor (EGF) and 0.25 % trypsin were purchased from Life Technologies (Carlsbad, California, USA). Penicillin, streptomycin, insulin and hydrocortisone were obtained from Sigma-Aldrich (Shanghai, China). The other materials used for cell culture were provided by Dr. Xiaofei Wang from the Institute of Animal Nutrition and Feed Science, Northwest A&F University, China.

Three healthy China Guanzhong dairy goats that had been raised in the livestock farm of Northwest A&F University since birth were selected for this study and used during the second parity and at peak lactation (day in milk (DIM) = 60 d). In detail, a 1 cm^3^ sample of the parenchymal tissue of the mammary gland was collected and placed in sterilized tubes containing ice-cold D-Hanks’ balanced salt solution (D-HBSS; pH = 7.4) after official approval for scientific sampling, and the tubes were immediately and aseptically transported to the laboratory immediately and aseptically. The tissue samples were washed with D-HBSS several times until the washing buffer was transparent, then sheared into 0.5 to 1.0 mm^3^ cubic fragments with a sterilized surgical scissor, and washed until clean. These fragments were placed in empty 60 mm cell culture dishes (Corning, New York, USA), maintaining an approximate distance of 0.5 cm between pieces, and the dishes were incubated in a cell incubator (Thermo Scientific, Massachusetts, USA) at 37 °C in 5 % CO_2_ and 95 % air for 30 min. Then, 1 mL of basal medium was added and incubated for 2 h, followed by the addition of another 1 mL of basal medium and incubation for an additional 48 h. The basal media contained 90 % DMEM/F-12 and 10 % FBS, and the concentrations of penicillin, streptomycin, insulin, hydrocortisone and EGF were 100.0 U/mL, 100.0 μg/mL, 5.0 μg/L, 1.0 μg/L and 1.0 μg/L, respectively. The medium was substituted for fresh basal medium every 48 h. When 90-95 % of the dish was occupied by visible cells under an inverted microscope (Nikon, Tokyo, Japan), the cells could be passaged. The cells were digested with 0.25 % trypsin for 5 min and passaged to new dishes. Subsequently, the medium was transferred to separate new culture plates 40 min later and incubated for 48 h to remove the fibroblasts. The adhesion time for fibroblasts (30 to 40 min) was shorter than that of MECs; hence, purified MECs were procured after the last procedure was repeated 5 times. The MECs were previously characterized by Wang et al. [22] in our college.

### Experimental design

Purified MECs passaged to 7–12 generations were used in this study. The cells were seeded in 24-well flat-bottom culture plates (Corning, New York, USA) at a density of 2.0 × 10^4^ cells per well. Afterward, 700 μL of basal medium was added to each well and incubated for 24 h. The medium was removed, the cells were washed with sterilized phosphate-buffered saline (PBS; pH = 7.4) 3 times, and then 700 μL/well of treatment medium containing 1,25-(OH)_2_D_3_ (Sigma-Aldrich, Shanghai, China) was added. The final concentrations of 1,25-(OH)_2_D_3_ in the medium were 0, 0.1, 1.0, 10.0 and 100.0 nmol/L, respectively. Each treatment was conducted on 6 replicates with 1 replicate per passage to avoid the potential effects of different passages. Culture dishes were incubated under the same conditions described above for 24 h, and then the subsequent steps and analyses were implemented.

A specific inhibitor of hexokinase 2 (HK2), 3-bromopyruvate (3-BrPA; Sigma-Aldrich, Shanghai, China), was added to the medium to investigate the potential effects of the cellular energy status on calcium transport. HK2 phosphorylates glucose to generate glucose-6-phosphate (G6P), the first step in the cellular glucose catabolism, and HK2 inhibition is usually used to study the effect of energy status on metabolic processes [23]. The concentrations of 1,25-(OH)_2_D_3_ were 0 or 10.0 nmol/L, and the 3-BrPA concentrations were 0 or 50.0 μmol/L, respectively. The other procedures were consistent with the 1,25-(OH)_2_D_3_ treatment.

### Cell proliferation measurement

A commercial 3-(4,5-dimethylthiazol-2-yl)-2,5-diphenyltetrazolium bromide (MTT) Kit was obtained from Jiancheng Bioengineering Institute (Nanjing, China) to measure cell proliferation. Briefly, the MECs were seeded in a 96-well plate (2.0 × 10^4^ cells/well; Corning, New York, USA) and were incubated with basal medium (200 μL/well) at 37 °C in 5 % CO_2_ and 95 % air for 24 h. Subsequently, the basal medium was replaced with treatment medium (150 μL/well) and incubated under standard conditions for 24 h. Then, 1× MTT (50 μL/well) was added and incubated under the same conditions for 4 h. The supernatant was removed carefully and 150 μL dimethyl sulfoxide (DMSO; Amresco, OH 44139, USA) was added to each well, followed by an 8 min mixing process using a Tablet Shaker (Kylin-Bell Lab Instruments Co., Ltd., Jiangsu, China). The absorbance at 570 nm was determined using a Microplate Reader (Power Wave XS2, Bio Tek, USA).

### Glucose determination

The glucose content in the medium was determined via a Glucose Assay Reagent Kit (Jiancheng, Nanjing, China) based on the glucose oxidase/peroxidase colorimetric method. Medium samples were collected in each well of culture dishes. The reaction reagent (1,000 μL) and liquid sample (10 μL) were mixed in a pure plastic tube, incubated at 37 °C for 15 min, and then the optical density (OD) at 505 nm was read on a Microplate Reader (Power Wave XS2, Bio Tek, USA). The OD of a tube with a standard glucose (Sigma-Aldrich, Shanghai, China) solution was determined using the same method as the test wells. The glucose concentration is presented in millimoles per liter (mmol/L).

### Total protein assay of MECs

The total protein content of the treated MECs was determined using a Coomassie Protein Assay Reagent (Jiancheng, Nanjing, China). The cells were lysed using a repeated freeze-thaw fragmentation method. Accordingly, the MECs were frozen at −80 °C for 60 min and transferred to a 37 °C water bath for 15 min to thaw the cells, which was repeated 3 times. Samples of the cell debris and contents were collected by adding 300 μL of a 0.9 % sodium chloride (NaCl) solution to each well. Double distilled water (blank control), a standard protein solution and sample liquid with an equal volume (50 μL) were mixed with 3.0 mL of reagent and incubated at room temperature for 10 min. Finally, the OD was recorded at a specific wavelength (595 nm) and optical path (1 cm) using a U3900 Spectrophotometer (Hitachi, Tokyo, Japan).

### Real Time Quantitative Polymerase Chain Reaction (RT-qPCR)

Total RNA was extracted from the MECs using an RNAprep Pure Cell/Bacteria Kit (TIANGEN, Beijing, China). The purity and concentration of the total RNA was determined using a NanoDrop 2000 UV–vis Spectrophotometer (Thermo Scientific, Massachusetts, USA). Reverse transcription was performed with a PrimeScript® RT reagent Kit (Takara Biotechnology, Dalian, China), and the cDNA samples were stored at −20 °C until further analysis. The mRNA expression levels of the facilitative Na^+^-independent glucose transporters (*GLUT1* and *GLUT12*), vitamin D receptor (*VDR*), *calbindin*-*D*_*9k*_, *PMCA1b*, *PMCA2b* and *Orai1* were measured using a SYBR® Premix Ex Taq™ II (Takara Biotechnology, Dalian, China). Briefly, a 20 μL reaction system was used that consisted of 10 μL of SYBR Premix Ex Taq II (2×), 0.8 μL of forward primer (10.0 μmol/L), 0.8 μL of reverse primer (10.0 μmol/L), 1 μL (500 ng) of cDNA, and 7.4 μL of RNase-free water. The reaction procedure was performed using an iCycler iQ5 multicolor real-time PCR detection system (Bio-Rad Laboratories, Hercules, CA) with the following program: 95 °C for 5 min; 35 cycles of 95 °C for 10 s, 60 °C for 30 s, and 72 °C for 30 s; and 72 °C for 5 min. All samples were run in triplicate, and the 2^-△△Ct^ method, which was previously established by Livak [24], was adopted to analyze the gene expression data. The primers are presented in Table 1, and β-actin was used as a reference gene in this study.

**Table 1:** Primer sequences used for the RT-qPCR analysis

Genes	Strand	Sequences (5′-3′)	Source
*β-actin*	Forward	CCTGCGGCATTCACGAAACTAC	JX046106.1
	Reverse	ACAGCACCGTGTTGGCGTAGAG	
*Calbindin*-*D*_*9k*_	Forward	TCTCCAGAAGAACTGAAGGGC	XM_005701057.2
	Reverse	CCAACACCTGGAATTCTTCG	
*GLUT1*	Forward	GCTAGCATGGAGCCCACCAGCAAG	JQ343217.1
	Reverse	AAGCTTTCACACTTGGGAATCAGCTCC	
*GLUT12*	Forward	GGAAAAGTGACCGCTCGTG	JQ798185.1
	Reverse	TGTCCTGGTAGGCAAAGAACTG	
*VDR*	Forward	GCACTTCCTTACCTGACCCC	[25]
	Reverse	CCGCTTGAGGATCATCTCCC	
*PMCA1b*	Forward	GAGACCATGGCTTGCTGAGT	[26]
	Reverse	GACCTTCTGGTACTGCCACC	
*PMCA2b*	Forward	GCATTTTCATCGGGTTAGGAG	[27]
	Reverse	AGAGCTACGAAACGCCTTCAC	
*Orai1*	Forward	CAGCGTGCATAATATACCTAACTCTACCCG	[28]
	Reverse	GTATTGATGAGGAGAGCAAGCGTGAAT	

### Western blot

After treatments, the supernatant fluid was removed and the cells were washed three times. Total protein was extracted using a High Performance RIPA buffer (Solarbio Science & Technology Co., Ltd., Beijing, China) in which the final concentration of phenylmethylsulfonyl fluoride (PMSF; Roche, Shanghai, China) was 1.0 mmol/L. The cells were collected in a 4 °C-precooled Eppendorf tube using a cell scraper, and the cells were lysed for 30 min at 4 °C. Afterward, the turbid liquid was centrifuged at a speed of 13,000 r/min for 10 min at 4 °C. The supernatant contained the total protein and was collected for further analysis. The western blot analysis was conducted according to the protocols reported by Xu et al. [29]. Briefly, the protein content was determined using a Pierce™ bicinchoninic acid (BCA) Protein Assay Kit (Thermo Scientific, Rockford, USA), according to the manufacturer’s instructions. The total proteins were separated by SDS-PAGE, transferred to nitrocellulose membranes (Millipore, Billerica, USA), and then probed with the primary antibodies anti-PMCA1b, anti-PMCA2b and anti-β-actin, which were all purchased from Abcam (Cambridge, UK). Goat anti-rabbit IgG (Abcam, Cambridge, UK) was used as a secondary antibody. The chemiluminescent ECL western blot assay system (Thermo, Rockford, USA) was used to detect the signals.

### Enzyme activity assay

A Hexokinase Test Kit (Jiancheng, Nanjing, China) was used to detect the HK activity of the solutions containing cell debris, and the samples were collected according to the user’s manual. The prepared reagent was pre-warmed at 37 °C for 10 min, and then 50 μL of liquid sample and 960 μL of reagent were immediately mixed in a tube to start the reaction. The absorbance at 340 nm (optical path: 0.5 cm) was recorded after 30 s (OD1) using a U3900 Spectrophotometer (Hitachi, Tokyo, Japan). Subsequently, the liquid was transferred back to the previous tube and warmed in a 37 °C water bath for 2 min. The absorbance was measured again under the same conditions and denoted as OD2. The HK activity was calculated using the following formula:

$$ \mathrm{HKactivity}\left(\frac{\mathrm{U}}{\mathrm{gprot}}\right)=\frac{\mathrm{OD}2-\mathrm{O}\mathrm{D}1}{6.22}\times \frac{1}{2\times 0.5}\times \frac{1.01}{0.05}\div \mathrm{C}\left(\mathrm{protein}\right) $$

where “6.22” represents the millimolar extinction coefficient, “2” represents the reaction time (min), “0.5” represents the optical path (cm), and “1.01/0.05” refers to the dilution factor.

The Na^+^K^+^-ATPase and Ca^2+^Mg^2+^-ATPase activities were detected with a Trace ATPase Test Kit (Jiancheng, Nanjing, China). Protein samples were mixed with the appropriate reagents (different reagents for these two enzymes) and heated in a 37 °C water bath for 10 min; then, another reagent was added to the reaction system and centrifuged at 3,500 r/min for 15 min. The supernatants were collected to determine the inorganic phosphate (Pi) concentration. The Pi samples were treated with the appropriate reagents at room temperature for 2 min. Afterward, a final reagent was added and incubated at room temperature for 5 min. The OD values at 636 nm (optical path: 1 cm), including blank control (OD_blank_), control (OD_control_), standard product (OD_standard_) and sample (OD_sample_), were read using a Microplate Reader (Power Wave XS2, Bio Tek, USA). The formula to determine the protein concentration is as follows:

$$ \mathrm{Enzymeactivity}\left(\mathrm{U}/\mathrm{mgprot}\right)=\frac{\mathrm{ODsample}-\mathrm{ODcontrol}}{\mathrm{ODstandard}-\mathrm{ODblank}}\times 0.02\times 6\times 7.8\div \mathrm{C}\left(\mathrm{protein}\right) $$

where “0.02” represents the concentration of the standard Pi solution (μmol/mL), “6” represents the reaction time (min), and “7.8” represents the dilution factor.

### Statistical analysis

The data were subjected to one-way analysis of variance (ANOVA) using Statistical Product and Service Solutions 21.0 (SPSS 21.0; IBM SPSS Statistics, USA), and multiple comparisons were performed using Duncan’s method [30]. The values were presented as the means ± SE (standard error). The results were declared significantly different if *P* < 0.05.

## Results

### Cell proliferation

**Fig. 1:**
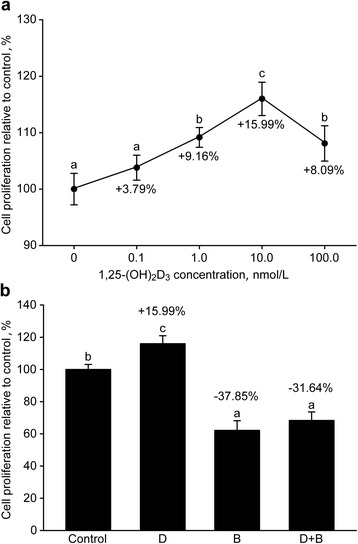
Proliferation of goat mammary epithelial cells in response to different 1,25-(OH)_2_D_3_ concentrations (**a**) and supplementation (**b**) with 1,25-(OH)_2_D_3_ (10.0 nmol/L) and 3-bromopyruvate (50.0 μmol/L) D = 1,25-Dihydroxyvitamin D_3_ (1,25-(OH)_2_D_3_, 10.0 nmol/L), B = 3-bromopyruvate (3-BrPA, 50.0 μmol/L), B + D = 3-BrPA plus 1,25-(OH)_2_D_3_. Different letters within a single figure represent a significant difference (*P* < 0.05)

Supplementation with 1,25-(OH)_2_D_3_ significantly promoted MEC proliferation as the concentration increased from 0.1 to 10.0 nmol/L (*P* < 0.05, Fig. 1a), and no difference was observed between the control and the 0.1 nmol/L group (*P* > 0.05). Compared with the control, the rates of cell proliferation at the concentration of 0.1, 1.0, 10.0 and 100.0 nmol/L were increased by 3.79 %, 9.16 %, 15.99 % and 8.09 %, respectively. The cell proliferation rate in the 100.0 nmol/L group (*P* < 0.05) was lower than the 10.0 nmol/L group. In addition, the proliferation rate in the 100.0 nmol/L group was statistically equal to the 1.0 nmol/L group (*P* > 0.05).

Cell proliferation was inhibited in the 3-BrPA-supplemented group and the 3-BrPA plus 1,25-(OH)_2_D_3_ group (*P* < 0.05, Fig. 1b), and proliferation decreased by 37.85 % and 31.64 %, respectively. Increased cell proliferation was observed in the 1,25-(OH)_2_D_3_ group without 3-BrPA supplementation (*P* < 0.05). Whether or not the 1,25-(OH)_2_D_3_ was supplemented, no difference was observed in the MECs treated with 3-BrPA (*P* > 0.05).

### Glucose consumption

**Fig. 2:**
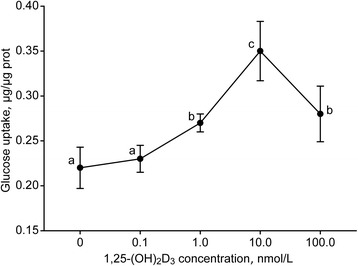
Glucose uptake of goat mammary epithelial cells in response to different 1,25-(OH)_2_D_3_ concentrations. Values with different letters were declared significant (*P* < 0.05)

The 0.1 nmol/L 1,25-(OH)_2_D_3_ treatment did not affect the glucose consumption by the goat MECs (*P* > 0.05, Fig. 2). The glucose uptake was significantly promoted when the 1,25-(OH)_2_D_3_ concentration increased from 0.1 to 10.0 nmol/L (*P* < 0.05). In accordance with cell proliferation, 100.0 nmol/L 1,25-(OH)_2_D_3_ decreased glucose consumption compared with the 10.0 nmol/L treatment (*P* < 0.05), and no differences were observed between 1.0 and 100.0 nmol/L (*P* > 0.05).

### Gene expression

**Fig. 3:**
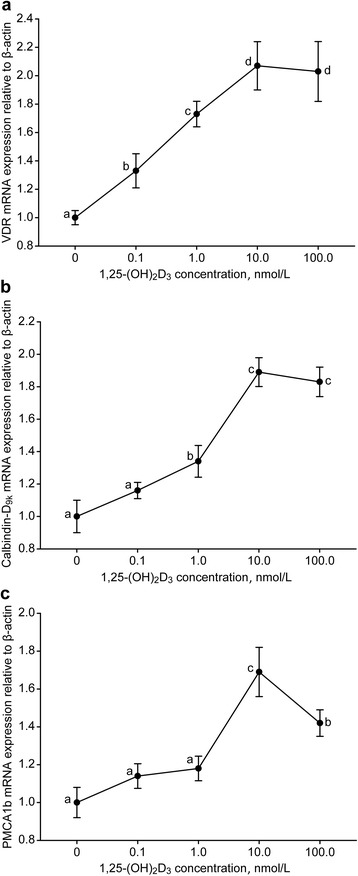
Expression of the vitamin D receptor (*VDR*), calcium binding protein D_9k_ (*Calbindin*-*D*_*9k*_) and plasma membrane Ca^2+^-ATPase 1b (*PMCA1b*) genes in goat mammary epithelial cells in response to different 1,25-(OH)_2_D_3_ concentrations. Different letters within a single figure represent a significant difference (*P* < 0.05)

The expression of genes related to calcium transport in goat MECs were presented in Fig. 3. An increase in *VDR* expression was observed as the 1,25-(OH)_2_D_3_ levels increased from 0 to 10.0 nmol/L (*P* < 0.05), whereas no effect was observed between 10.0 and 100.0 nmol/L (*P* > 0.05). The same trend was observed for *calbindin*-*D*_*9k*_, with the exception of an insignificant difference at 0.1 nmol/L compared with the control. In addition, supplementation with 10.0 and 100.0 nmol/L 1,25-(OH)_2_D_3_ increased *PMCA1b* expression (*P* < 0.05), and the peak *PMCA1b* expression level appeared at 10.0 nmol/L (*P* < 0.05). However, 1,25-(OH)_2_D_3_ had no influence on *PMCA1b* expression at concentrations of 0 and 1.0 nmol/L (*P* > 0.05).

**Fig. 4:**
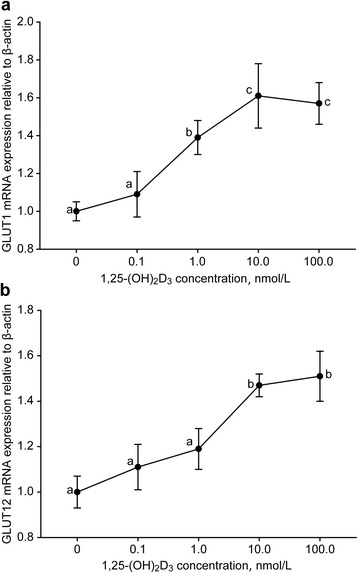
Expression of the facilitative Na^+^-independent glucose transporter (*GLUT1* and *GLUT12*) genes in goat mammary epithelial cells in response to different 1, 25-(OH)_2_D_3_ levels. Different letters within a single figure represent a significant difference (*P* < 0.05)

The 1,25-(OH)_2_D_3_ supplementation altered the *GLUT1* and *GLUT12* gene expression levels as well (Fig. 4). There was an increase in *GLUT1* mRNA abundance as the 1,25-(OH)_2_D_3_ levels increased from 0.1 to 10.0 nmol/L (*P* < 0.05, Fig. 4a). No difference was observed between the control and 0.1 nmol/L. However, compared with 10.0 nmol/L 1,25-(OH)_2_D_3_, the 100 nmol/L treatment did not increase *GLUT1* expression (*P* > 0.05). Inconsistently, supplementation with 1,25-(OH)_2_D_3_ had no influence on *GLUT12* expression when the concentration was less than 1.0 nmol/L (*P* > 0.05, Fig. 4b). The 10.0 nmol/L treatment promoted *GLUT12* expression compared to the 1.0 nmol/L treatment (*P* < 0.05), and there was no difference between the 10.0 and 100.0 nmol/L treatments (*P* > 0.05).

**Fig. 5:**
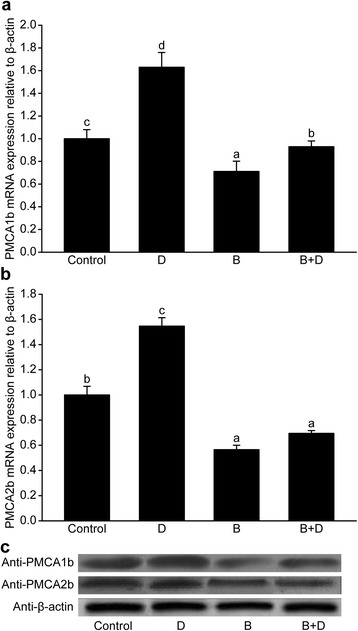
Expression of the plasma membrane Ca^2+^-ATPase 1b (*PMCA1b*, A) and 2b (*PMCA2b*, B) genes and representative immunoblots (C) of PMCA1b, PMCA2b and β-actin in goat mammary epithelial cells in response to supplementation with 1,25-(OH)_2_D_3_ (10.0 nmol/L) and 3-bromopyruvate (3-BrPA, 50.0 μmol/L). D = 1,25-Dihydroxyvitamin D_3_ (1,25-(OH)_2_D_3_, 10.0 nmol/L), B = 3-bromopyruvate (3-BrPA, 50.0 μmol/L), B + D = 3-BrPA plus 1,25-(OH)_2_D_3_. Different letters within a single figure represent a significant difference (*P* < 0.05)

Supplementation with 3-BrPA down-regulated *PMCA1b* and *PMCA2b* expression (*P* < 0.05, Fig. 5a and b), regardless of whether 1,25-(OH)_2_D_3_ was added. The expression levels of *PMCA1b* and *PMCA2b* in group D (10.0 nmol/L 1,25-(OH)_2_D_3_) were higher than those of the control. Specifically, the 1,25-(OH)_2_D_3_ treatment up-regulated *PMCA1*b expression in the 3-BrPA-supplemented groups (*P* < 0.05, Fig. 5a), but no difference in *PMCA2b* expression was observed (*P* > 0.05, Fig. 5b). As we could see from the immunoblots (Fig. 5c), the changes in the levels of the PMCA1b and PMCA2b proteins in the supplemented groups were similar to the changes in the transcripts.

**Fig. 6:**
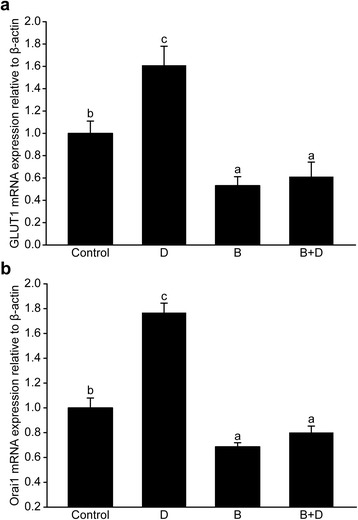
Expressions of the facilitative Na^+^-independent glucose transporter1 (*GLUT1*, **a**) and *Orai1* genes (**b**) in goat mammary epithelial cells in response to supplementation with 1,25-(OH)_2_D_3_ (10.0 nmol/L) and 3-bromopyruvate (3-BrPA, 50.0 μmol/L). D = 1,25-Dihydroxyvitamin D_3_ (1,25-(OH)_2_D_3_, 10.0 nmol/L). Different letters within a single figure represent a significant difference (*P* < 0.05)

As shown in Fig. 6, the expression levels of *GLUT1* and *Orai1* were increased by 1,25-(OH)_2_D_3_ supplementation (*P* < 0.05) and reduced by the addition of 3-BrPA (*P* < 0.05). No difference was observed between the 3-BrPA-supplemented group and 1,25-(OH)_2_D_3_ plus 3-BrPA group (*P* > 0.05).

### Cell metabolic enzymes

As a whole, the enzyme activities, including HK, Ca^2+^Mg^2+^-ATPase and Na^+^K^+^-ATPase, were increased when the 1,25-(OH)_2_D_3_ levels increased from 0 to 10.0 nmol/L (Table 2). Compared with the 10.0 nmol/L treatment, decreased activities were detected in the 100.0 nmol/L group (*P* < 0.05). The HK activity in the 100.0 nmol/L group was statistically equal to the 0.1 nmol/L and control groups (*P* > 0.05). Supplementation with 0.1 nmol/L 1,25-(OH)_2_D_3_ did not affect the Ca^2+^Mg^2+^-ATPase and Na^+^K^+^-ATPase activities (*P* > 0.05), and no difference in Ca^2+^Mg^2+^-ATPase activity was observed between the 0.1 and 1.0 nmol/L groups (*P* > 0.05). The Na^+^K^+^-ATPase activity in the 100.0 nmol/L group was equivalent to the control (*P* > 0.05). Moreover, the Ca^2+^Mg^2+^-ATPase activity presented a sudden decrease at the highest 1,25-(OH)_2_D_3_ concentration, which was even lower than the control (*P* < 0.05).

**Table 2:** Effect of the 1,25-(OH)_2_D_3_ concentration on the metabolic enzyme activities in goat mammary epithelial cells

Item	1,25-(OH)_2_D_3_ concentration, nmol/L				
	0	0.1	1.0	10.0	100.0
Hexokinase, U/gprot	74.92 ± 1.25^a^	78.02 ± 1.92^a^	83.37 ± 1.73^b^	89.52 ± 2.14^c^	77.72 ± 1.59^a^
Ca^2+^Mg^2+^-ATPase, U/mgprot	0.71 ± 0.03^b^	0.78 ± 0.04^bc^	0.85 ± 0.07^c^	0.96 ± 0.11^d^	0.62 ± 0.09^a^
Na^+^K^+^-ATPase, U/mgprot	1.47 ± 0.07^a^	1.54 ± 0.12^ab^	1.63 ± 0.09^b^	1.81 ± 0.12^c^	1.46 ± 0.11^a^

^a-d^Superscripts with varied letters in the same row were significantly different (*P* < 0.05). Values are presented as the Means ± SE (standard error)

## Discussion

1,25-Dihydroxyvitamin D_3_ is a natural ligand of the vitamin D receptor (VDR), and plays an important role in anti-inflammatory processes and calcium transport [31, 32]. It has been reported that 1,25-(OH)_2_D_3_ could activate the VDR to modulate gene transcription and mineral ion homeostasis [33, 34]. Vitamin D-facilitated calcium transport is a complicated process, including the up-regulation and down-regulation of associated genes. Calbindin-D_9k_, PMCAs and Orai were considered essential elements for transcellular calcium transport following stimulation with 1,25-(OH)_2_D_3_ [10, 11, 35, 36]. Our data showed that 1,25-(OH)_2_D_3_ influenced the expression of the *VDR*, *calbindin*-*D*_*9k*_, *PMCA1b*, *PMCA2b* and *Orai1* genes in goat MECs in a dose-dependent manner, which indicated enhanced calcium transport. Furthermore, we could infer that this process was closely related to cellular energy availability, based on the changes in *GLUT1* and *GLUT12 *expression and the responses after the inhibition of HK2.

Supplementation with 1,25-(OH)_2_D_3_ improved cell proliferation in a concentration-dependent manner, with the exception of a relative decrease at 100.0 nmol/L. Our results were inconsistent with the results reported by Rayalam et al. [37], who found that 1,25-(OH)_2_D_3_ enhanced preadipocyte viability generated from 3 T3-L1 mouse embryo fibroblasts in a dose-dependent manner from 0.1 to 10.0 nmol/L, but no significant difference existed between the 10.0 and 100.0 nmol/L treatments. However, the proliferation of human airway smooth muscle cells (HASMCs) was gradually inhibited by increasing levels of 1,25-(OH)_2_D_3_ in another experiment [38]. These variant effects might result from different cell types and functions as well as from the tolerated doses. Due to the high calcium content of milk, MECs assimilate large amounts of calcium from plasma. In addition, calcium is an essential element for cell growth, differentiation and maintenance. Consequently, it is plausible that the 1,25-(OH)_2_D_3_-induced promotion of calcium uptake can enhance MECs proliferation. To our knowledge, this was the first study in which 1,25-(OH)_2_D_3_-stimulated cell proliferation of secreting MECs was investigated.

Mammary lactation is a complicated biological process that is sustained by a variety of nutrients, among which glucose acts as the supreme precursor for lactose synthesis as well as an energy resource of metabolic activities [23]. Hence, glucose plays an essential role in mammary milk secretion. It has been testified that glucose transporters (GLUTs) are the main tools for glucose uptake by mammary epithelial cells, and GLUT1 was the major transporter, although GLUT12 is involved as well [23, 39]. Previous studies rarely called attention to the effects of 1,25-(OH)_2_D_3_ on glucose uptake and metabolism. In our present study, 1,25-(OH)_2_D_3_ increased cell glucose consumption and up-regulated *GLUT1* and *GLUT12 *expression, indicating that more glucose was utilized for cell metabolism or component synthesis. In addition, intracellular glucose phosphorylation catalyzed by HK is the first step in energy metabolism and is a rate-limiting process. Consequently, the increased HK activity was another persuasive indicator of glucose utilization [23, 40]. The main reason for the enhanced glucose consumption might be that 1,25-(OH)_2_D_3_-induced calcium transport led to the promotion of milk secretion in goat MECs. In addition, several studies have shown that 1,25-(OH)_2_D_3_ regulated the immune response in ruminants [41–43], which also required energy to sustain the process.

1,25-(OH)_2_D_3_ is a flexible secosteroid and exerts its regulatory functions by binding to VDR, a specific nuclear receptor and DNA-binding transcription factor [44]. A series of biological processes, such as maintaining calcium homeostasis and mediating inflammation responses, are triggered by the binding between ligand and receptor [45]. We found that 0 to 10.0 nmol/L 1,25-(OH)_2_D_3_ promoted *VDR* expression, with no difference between the 10.0 and 100.0 nmol/L treatments. This finding indicated that 1,25-(OH)_2_D_3_ could increase the number of VDRs in a dose-dependent manner, with an optimal concentration of 10.0 nmol/L. Haussler et al. [44] noted that the activation and function of VDR were induced by 1,25-(OH)_2_D_3_, but saturation was not mentioned. From the authors’ point of view, the cell metabolic capacity was limited and could not be induced in an unlimited manner. This hypothesis was supported by the results from a previous study by Rayalam et al. [37], who discovered that 1,25-(OH)_2_D_3_ could no longer promote adipocyte growth when the concentration exceeded 10.0 nmol/L.

The diffusion of intracellular calcium from the apical side to basolateral side depends on its binding to calbindin-D_9k_, and calcium passes through the basolateral side via PMCA1b [1, 5, 9, 10]. An overall increase in the calbindin-D_9k_ and PMCA1b transcripts was detected when the 1,25-(OH)_2_D_3_ concentrations ranged from 0 to 10.0 nmol/L, which was a marker to distinguish the enhanced calcium transport. According to previous findings, both calbindin-D_9k_ and PMCA1b had a vitamin D response element (VDRE) in their promoter region, and the VDRE was the direct binding site of VDR [37, 46, 47], which may be why 1,25-(OH)_2_D_3_ could regulate transcellular calcium transport. Moreover, there are other proteins that regulate cellular calcium transport. Using a null mutation mouse model, Reinhardt et al. [48] showed that the activity of PMCA2b, another isoform of PMCA, was required for the secretion of milk calcium, and Ji et al. [17] showed that 1,25-(OH)_2_D_3_ could stimulate PMCA2b expression to regulate mammary calcium transport. Davis et al. [28] suggested that Orai1, a novel channel, was important for mammary calcium transport during lactation. Orai1 is a key component of the CRAC channels and plays an extremely important role in the transmembrane influx of calcium [13, 14, 36]. The biology and molecular mechanism of Orai1 have been reviewed by Cahalan et al. [12] and Hogan et al. [49]. The 1,25-(OH)_2_D_3_-stimulated up-regulation of PMCA2b and Orai1, together with their down-regulation by the inhibition of glucose metabolism, indicated that calcium transport in goat MECs could be regulated by 1,25-(OH)_2_D_3_ availability and the cellular energy status.

Plasma membrane Ca^2+^-ATPase is a transcellular Ca^2+^ transporter encoded by the *PMCA* gene family that plays a vital role in regulating cellular calcium metabolism and maintaining intracellular Ca^2+^ homeostasis [28, 50]. Ca^2+^Mg^2+^-ATPase activity showed a similar trend as the expression of *PMCA1b* and *PMCA2b*, indirectly indicating that calcium secretion was promoted when the 1,25-(OH)_2_D_3_ concentration did not exceed 10.0 nmol/L. Additionally, there was recent evidence showing that Na^+^/Ca^2+^ exchangers (NCX) on the mammalian plasma membrane co-modulated calcium transport with PMCA [50, 51]. Moreover, Zanatta et al. [52] found that 1,25-(OH)_2_D_3_ mediated transcellular calcium transport by stimulating NCX activation in rat Sertoli cells. Our data also showed an increase in Na^+^K^+^-ATPase activity as the 1,25-(OH)_2_D_3_ levels increased from 0 to 10.0 nmol/L. However, NCX expression was not examined in this study; therefore, we could not verify its regulatory role in the Ca^2+^ transcellular transport process.

Previous studies showed that 3-BrPA inhibited glycolysis in a dose-dependent manner by decreasing HK activity, particularly HK2; thus it has been widely used to investigate the impact of cellular energy status on biological processes [53, 54]. In our trials, the effect of energy availability on calcium transport in goat MECs was studied by supplementing the cells with 3-BrPA. Accordingly, cell proliferation and *GLUT1* expression decreased, which was most likely due to the inhibition of glucose metabolism. In support of our findings, Yun et al. [53] described that glycolysis inhibitors, such as 3-BrPA, could inhibit cell and tumor growth at proper dosages. The decrease in PMCA1b and PMCA2b expression at the mRNA and protein levels, as well as down-regulated Orai1 transcription, attested that calcium transport was inhibited in goat MECs. Hence, 1,25-(OH)_2_D_3_ promoted calcium transport in goat MECs, and this process depended on the intracellular availability of glucose. It is well known that glucose is the main energy source of many metabolic activities, and active nutrient transport is a process that expends energy. Therefore, the inhibition of glycolysis reduced *PMCA* and *Orai1* expression.

Compared with the 3-BrPA group, the 3-BrPA plus 1,25-(OH)_2_D_3_ group exhibited higher *PMCA1b* expression, whereas *GLUT1* expression showed no difference, indicating that 1,25-(OH)_2_D_3_ could still enhance calcium transport when glucose uptake was suppressed in goat MECs. To our knowledge, this was a novel discovery. Many substances, such as clenbuterol [55] and conjugated linoleic acids (CLAs) [56], have been proven to induce nutrient repartition. We speculated that the stimulation of 1,25-(OH)_2_D_3_ repartitioned cellular energy for calcium secretion, but this assumption required convincing support. More trials are required to explore the roles of PMCAs, Orai1, NCX and other potential proteins. From the authors’ point of view, mammary calcium secretion is a complicated system, and multiple, cross-linked networks should be established via transcriptomics and proteomics technologies to better understand milk calcium synthesis. In addition, the isotope tracer technology should be used to directly reflect mammary calcium transport in dairy goats.

## Conclusions

Suitable concentrations of 1,25-(OH)_2_D_3_ promoted proliferation and glucose utilization in goat MECs in a dose-dependent manner. Supplementation with 1,25-(OH)_2_D_3_ could modulate calcium transport by altering the expression of *VDR*, *calbindin*-*D*_*9k*_, *PMCA1b*, *PMCA2b* and *Orai1* in a dose- and energy-dependent manner. In the present study, the optimal concentration of 1,25-(OH)_2_D_3_ that stimulated the expression of calcium transport indicators in goat MECs was 10.0 nmol/L. Our findings highlighted the role of 1,25-(OH)_2_D_3_ as a potential regulatory agent to produce calcium-enriched milk in ruminants when sufficient intracellular energy was available.
